# Hypersensitivity Reactions to Benzodiazepines Used for Perioperative Premedication: A Narrative Review

**DOI:** 10.3390/ph19030507

**Published:** 2026-03-20

**Authors:** Julia Gąsiorowska, Emilia Kiełczyńska, Weronika Dziamara, Amelia Grundys, Justyna Drozdowska, Monika Woźny, Aleksandra Skiba, Krzysztof Gomułka

**Affiliations:** 1 Student Research Group of Allergology and Internal Medicine, Wroclaw Medical University, 50-556 Wrocław, Poland; emilia.kielczynska@student.umw.edu.pl (E.K.); weronika.dziamara@student.umw.edu.pl (W.D.); amelia.grundys@student.umw.edu.pl (A.G.); justyna.drozdowska@student.umw.edu.pl (J.D.); monika.wozny@student.umw.edu.pl (M.W.); aleksandra.skiba@student.umw.edu.pl (A.S.); 2 Clinical Department of Allergology and Internal Medicine, Wroclaw Medical University, 50-556 Wrocław, Poland; krzysztof.gomulka@umw.edu.pl

**Keywords:** benzodiazepines, hypersensitivity, allergic reactions, midazolam, remimazolam, lorazepam

## Abstract

**Background**: Hypersensitivity reactions to benzodiazepines, although uncommon, represent a clinically relevant issue in perioperative practice. Benzodiazepines are widely used medications with anxiolytic, sedative, and anticonvulsant properties. The objective of this review is to synthesize current knowledge on benzodiazepine hypersensitivity, focusing on underlying mechanisms, clinical manifestations, diagnostic considerations, and strategies for management and prevention in the perioperative setting. **Methodology**: A narrative synthesis of the current literature on hypersensitivity reactions to benzodiazepines was performed. The review included published case reports, case series, and clinical studies describing hypersensitivity reactions, their clinical presentation, and diagnostic approaches. Particular attention was given to both immunological and non-immunological mechanisms, reported clinical phenotypes, and issues relevant to perioperative patient safety. **Results**: The extant evidence suggests that benzodiazepine hypersensitivity may involve both immunological and non-immunological pathways. The spectrum of reported reactions encompasses mild cutaneous manifestations and severe systemic responses, although the incidence remains low. This review highlights diagnostic challenges related to variable clinical presentation and the limited availability of standardized testing methods. **Conclusions**: Although cases of benzodiazepine hypersensitivity are uncommon, awareness of potential reactions is critical for ensuring safe clinical practice. This review emphasizes the necessity for additional research to elucidate the underlying mechanisms, standardize the diagnostic criteria, and formulate management protocols.

## 1. Introduction

Premedication in the perioperative setting aims to reduce anxiety and ensure patient comfort before the induction of anesthesia. Benzodiazepines play a central role in this process by providing effective anxiolysis and sedation while helping to minimize stress-related adverse reactions [1,2,3].

Benzodiazepines represent one of the most commonly used drug classes for perioperative premedication, with midazolam, diazepam, lorazepam, and remimazolam being the most frequently administered agents. Dosing should be individualized based on the patient’s clinical condition, the type of procedure, and the available monitoring capabilities. The route of administration is chosen to optimize patient comfort, with several options, including sublingual, oral, intranasal, rectal, intravenous, and intramuscular delivery [4,5]. Despite their well-established safety profile, benzodiazepines may, in rare instances, induce hypersensitivity reactions.

Within the broader epidemiological spectrum of perioperative anaphylaxis, neuromuscular blocking agents, antibiotics, latex, and chlorhexidine represent the most commonly implicated triggers. Hypersensitivity reactions to benzodiazepines presenting as preoperative anaphylaxis are rare but have been documented in the literature. The reported incidence of anaphylaxis caused by benzodiazepines ranges from approximately 1 in 35,000 to 1 in 20,000 cases. Despite their low frequency, such reactions are clinically relevant because of their potentially life-threatening nature and risk of fatal outcomes. Therefore, awareness of this perioperative risk remains essential [6].

In the perioperative setting, where multiple pharmacological agents are often administered within a short temporal window, identification of the causative compound poses a substantial diagnostic challenge. Benzodiazepines require particular attention because their early administration, often before full physiological monitoring is established, can delay recognition and attribution of hypersensitivity reactions, making it difficult to distinguish these reactions from other perioperative events. Furthermore, their distinct chemical structure and receptor-mediated mechanisms of action differentiate them from other major perioperative allergens, potentially influencing immunopathogenic pathways and cross-reactivity profiles. Despite these considerations, benzodiazepines remain underrepresented in structured analyses of perioperative hypersensitivity, and current evidence is largely confined to isolated case reports and small case series.

Although hypersensitivity reactions to benzodiazepines are uncommon, they remain clinically relevant and should be considered, particularly in patients exposed to polypharmacy, with organ dysfunction, or with a history of drug-induced hypersensitivity [7,8]. Accurate documentation of the suspected agent administered dose, latency to symptom onset, clinical phenotype, comorbidities, and concurrent medications is essential. Diagnostic evaluation may include skin testing, specific IgE assays, basophil activation tests, and controlled drug provocation tests.

When hypersensitivity is confirmed, clinical management aims to (1) identify a safe and effective alternative agent, (2) ensure adequate preparation for subsequent procedures, and (3) minimize the risk of cross-reactivity. In circumstances requiring benzodiazepine administration, particularly in high-risk patients, enhanced perioperative surveillance and consideration of alternative premedication strategies, such as α2-adrenergic receptor agonists, antihistamines, or antiemetics, are recommended in accordance with contemporary perioperative hypersensitivity guidelines [9,10].

In clinical practice, careful assessment of a patient’s history of prior allergic or anaphylactic reactions is recommended before administering benzodiazepines.

This review provides a concise synthesis of current knowledge regarding benzodiazepine-induced hypersensitivity, focusing on clinical manifestations, diagnostic strategies, and therapeutic and preventive approaches to enhance patient safety in perioperative care.

## 2. Results

### 2.1. The Most Used Benzodiazepines in Premedication

Benzodiazepines are commonly used as premedication prior to surgical procedures. The most frequently administered benzodiazepines to reduce anxiety, decrease muscle tension, and induce anterograde amnesia include midazolam, diazepam, lorazepam, and the more recently introduced remimazolam. Although these drugs are generally considered safe, hypersensitivity reactions have been described. However, the available evidence remains limited, with most data derived from single-case reports and small case series involving midazolam and the relatively new remimazolam [11].

#### 2.1.1. Midazolam

An analysis of patient cases demonstrated that hypersensitivity reactions to midazolam predominantly manifested as cardiovascular symptoms, local cutaneous reactions at the injection site, and systemic reactions suggestive of an IgE-mediated mechanism, with intradermal test positivity and elevated tryptase levels supporting mast cell activation [5,12]. In one case, a patient exhibited symptoms of bradycardia, accompanied by the presence of wide QRS complexes and ST segment elevation on electrocardiographic (ECG) analysis. This was followed by a decline in both heart rate and blood pressure, resulting in asystole, a condition that necessitated resuscitation measures [5].

Local reactions included urticarial rash and erythema at the site of intravenous midazolam injection. Symptoms resolved following administration of intravenous corticosteroids and antihistamines, without requiring resuscitative intervention [5].

Systemic symptoms were also observed, including nausea, epigastric pain, extensive erythematous rash, and pruritus. In one case, laryngeal edema and dyspnea also occurred, indicating a severe anaphylactic reaction [5].

#### 2.1.2. Remimazolam

The currently available evidence regarding hypersensitivity reactions to remimazolam is limited and derives primarily from isolated case reports. In these reports, reactions occurred rapidly after administration and included cutaneous, respiratory and hemodynamic manifestations.

Initial symptoms described in individual cases included facial flushing, periorbital and perioral edema, and a extensive exanthema developing within minutes of the initiation of remimazolam administration [11,13]. Some patients developed laryngeal stridor and epiglottic edema, resulting in decreased SpO_2_ and necessitating intensive respiratory intervention [14]. Hemodynamic manifestations included hypotension and tachycardia, with one case progressing to cardiac arrest that required resuscitation [11].

Given that these observations originated from isolated clinical reports, they should be interpreted with caution and cannot be considered representative of the full clinical spectrum of remimazolam hypersensitivity.

Midazolam and remimazolam belong to the imidazobenzodiazepine class and share considerable structural similarity, which suggests a theoretical risk of cross-reactivity between these agents [13]. However, the available clinical evidence regarding such cross-reactivity remains sparse and inconsistent. In isolated case reports, skin tests demonstrated simultaneous positive reactions to both midazolam and remimazolam, suggesting the possibility of prior sensitization to midazolam and subsequent anaphylaxis following administration of remimazolam [13]. In other reports, cross-reactivity was not confirmed, and midazolam was safely administered both before and after the episode of remimazolam-induced anaphylaxis [11,15].

An additional factor complicating the interpretation of skin test results is the presence of dextran 40 in the remimazolam formulation, which can trigger non-IgE-mediated anaphylactic reactions and lead to false-negative test results [11,15]. Consequently, anaphylaxis following remimazolam administration may occur either independently of hypersensitivity to midazolam or—less commonly—via a cross-reactivity mechanism [13]. In clinical practice, the decision to use an alternative benzodiazepine after a reaction to remimazolam should be made on an individual basis, guided by detailed allergy testing that includes remimazolam, midazolam, and, in selected cases, dextran 40 [11,15].

### 2.2. Hypersensitivity Mechanisms

Adverse reactions to benzodiazepines are diverse and must be strictly categorized to maintain diagnostic accuracy. Hypersensitivity reactions are classified into allergic hypersensitivity (mediated by immunological mechanisms, such as IgE antibodies or T cells) and non-allergic hypersensitivity (termed as pseudoallergy), involving non-immune mechanisms.

**Figure 1:**
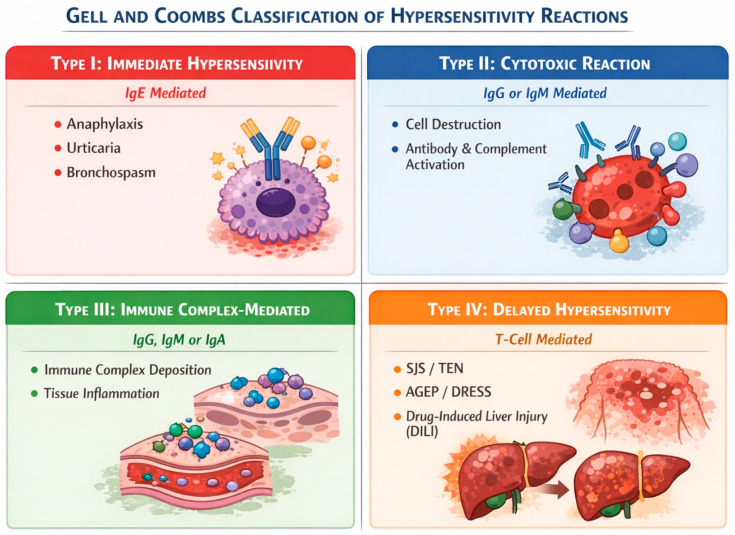
Characteristics of hypersensitivity classification according to Gell and Coombs. The figure was created using Canva (https://www.canva.com, accessed on 20 December 2025).

Immunological reactions are heterogeneous, involving the activation of the immune system via different pathways, leading to various clinical manifestations. As illustrated in Figure 1, these reactions are categorized according to the Gell and Coombs classification.

Type I reactions (Immediate): Mediated by IgE antibodies that bind to mast cells or basophils, triggering the release of mediators that cause anaphylaxis, urticaria and bronchospasm. Notably, an IgG-mediated anaphylaxis has also been documented [16].
Type II reactions (Cytotoxic): Mediated by drug-specific IgG or IgM antibodies. Cell destruction results from direct antibody–cell interaction or complement activation.
Type III reactions (Immune Complex): Mediated by IgG, IgM or IgA deposition in tissue leading to organ injury.
Type IV reactions (Delayed): These T cell-mediated reactions typically present with a delayed onset. This category includes severe cutaneous adverse reactions (SCAR), such as SJS/TEN, AGEP, and DRESS. The reaction may also manifest as single-organ involvement, most commonly drug-induced liver injury (DILI) [16,17].

In the context of benzodiazepines, immunological reactions may be described through specific molecular mechanisms [16,17].

The hapten model

This model describes an IgE-mediated reaction, occurring when a low-molecular-weight drug or its metabolites bind covalently to host proteins, forming an antigen–protein complex. Haptens are small chemically active molecules that are not immunogenic on their own but can become so upon binding to proteins. It may lead to the generation of drug-specific humoral or cellular immune responses. These complexes are presented by antigen-presenting cells (APC) in association with MHC I or MHC II molecules, potentially leading to T-cell activation.

IgE-mediated benzodiazepine hypersensitivity is rare; however, an increasing number of case reports suggests that this condition may be underrecognized.

The danger model

Rather than representing a distinct clinical classification, the danger model serves as a prerequisite mechanism for true immunological sensitization. It posits that while the drug itself is often insufficient to cause a reaction, the status of the immunological system is crucial to suppressing the hypersensitivity reaction. The coincidence of drug administration and a compromised immunological host status, such as physical stress or viral infection, may trigger the immune system resulting in non-HLA-restricted drug allergies.

Pharmacological interaction with immune receptors (P–I model reaction)

This model explains idiosyncratic reactions. It involves a noncovalent bond between the drug in its native form and immune receptors, such as the T-cell receptor (TCR) or HLA, without the need for peptide processing. The interaction leads to T-lymphocyte activation. In certain cases, drugs used in anesthesia may trigger T-cell responses in the absence of intracellular peptide processing may occur.

Pharmacogenetic reactions

These reactions are often rooted in both P–I and heterologous immunity models. Genetic predisposition, specifically the presence of specific HLA alleles, may influence the capability of the drug to interact interacting with TCR or HLA molecules and mediate a T-cell response. The heterologous immunity model suggests that the presence of an HLA risk allele, prior infection by a pathogen, and pathogen-specific memory T cells are involved in developing adverse drug reactions (ADR).

The altered self-repertoire model

An interesting altered self-repertoire model also has been described. Drug molecules accommodate noncovalently by binding grooves of an HLA molecule altering its conformation. This alters the repertoire of self-peptide ligands bound and presented by APC’s, potentially leading to a primary, polyclonal immune response mediated by T cells.

The fundamental models of drug hypersensitivity involve mechanisms such as haptenization or the pharmacological interaction with immune receptors (p–i model), where drugs bind noncovalently to HLA or TCR. While these pathways are well-described for many compounds, their role in benzodiazepine-induced reactions remains largely theoretical and has not yet been definitively proven. Furthermore, it remains unclear why only a small subset of patients carrying risk alleles develops clinical symptoms or why different drug–HLA binding configurations result in different clinical syndromes. Furthermore, some drugs may trigger immune stimulation by concurrently engaging multiple pathways, including hapten-mediated, p–I, and/or pseudo-allergic mechanisms [18]

To evaluate perioperative anaphylaxis reaction the Ring and Messmer four-step grading scale is used in Table 1 [19].

Perioperative hypersensitivity remains an important and actively explored area of research. Although some systemic reactions have been clinically associated with histamine release potentially triggered by benzodiazepines, solid pharmacological evidence for direct mast cell activation by these agents is lacking. Non-immunologic pathways remain largely speculative and may involve indirect mechanisms, whereas IgE-mediated reactions, although rare, have been documented in both original studies and case reports.

Both prospective and retrospective clinical studies analyzing anesthetic data from previous surgical procedures were systematically evaluated, as presented in Table 2. Skin testing, including epidermal skin prick tests (SPT) and intradermal tests (IDT) was the “diagnostic gold standard” used across the analyzed studies. In some investigations, basophil activation tests (BAT) or serological assays were additionally performed [20].

In the analyzed retrospective studies investigating drug-induced perioperative anaphylaxis, the diagnostic yield for intravenous midazolam varied significantly depending on the cohort selection. The frequency of confirmed reactions attributed to intravenous midazolam in adult patients ranged from 13.8% [21] to 35.7% [4]. It is imperative to distinguish these figures from population-wide incidence rates. These percentages do not represent the incidence in the general surgical population but the proportion of confirmed midazolam allergies among patients already referred for specialized workup due to suspected perioperative reaction. For instance, the higher rate (35.7%) was calculated within a specific subgroup of patients selected for midazolam testing (10/28), whereas the lower estimate refers to the broader cohort evaluated for perioperative hypersensitivity. In the pediatric population, a retrospective study reported a lower rate, where midazolam accounted for 6% of perioperative anaphylaxis. [22]. This rate was calculated as the ratio of diagnostically confirmed midazolam reactions to the total number of patients evaluated for suspected perioperative hypersensitivity in the study cohort. Consequently, these figures represent diagnostic yields within specialized allergy-center cohorts and do not reflect the true population-wide incidence. The clinical presentations across these studies primarily involved the cardiovascular, respiratory, and cutaneous systems.

Available reports suggest that the majority of documented hypersensitivity reactions to benzodiazepines used in premedication and anesthesia align with IgE-mediated (Type I) mechanisms. However, this observation is based primarily on limited case reports and small study cohorts, which precludes a definitive conclusion regarding the overall dominance of this mechanism. While non-immunologic (pseudoallergic) reactions appear to be reported less frequently, their etiology often remains undetermined. Despite the limited scale of current evidence, these adverse events pose a significant clinical challenge owing to their potential severity.

### 2.3. Paradoxical Reactions Occurring After the Use of Benzodiazepines

Paradoxical reactions represent atypical and unpredictable responses to benzodiazepines characterized by excitatory neuropsychiatric manifestations, such as agitation, confusion, delirium, aggressive behavior, restlessness, and involuntary movements, occurring instead of the expected anxiolytic and sedative effects. These reactions may develop rapidly after administration, including following standard premedication doses of midazolam used for perioperative anxiety [23,24].

Importantly, paradoxical reactions are considered pharmacodynamic effects rather than hypersensitivity reactions. However, their clinical presentation may mimic agitation associated with other adverse drug reactions, which may complicate differential diagnosis in the perioperative setting.

In large cohorts of sedated endoscopic procedures, paradoxical reactions have been reported in approximately 0.86% of cases, with a recurrence rate of 30.7% among patients with a prior history of such reactions [25]. Misinterpretation of paradoxical agitation as inadequate sedation may lead to additional benzodiazepine administration, which can exacerbate symptoms rather than provide clinical improvement. In some cases, rapid resolution following flumazenil administration supports a benzodiazepine-specific pharmacological mechanism [24,26].

### 2.4. Clinical Picture

The clinical presentation of benzodiazepine hypersensitivity is highly variable, ranging from mild skin manifestation, such as urticaria, to severe reactions, including anaphylactic or anaphylactoid reactions. Both IgE-mediated and non-IgE-mediated reactions may present with a similar clinical picture. In the analyzed reports, the onset of symptoms ranged from 1 min to an 8 h after drug administration, with the majority of reactions occurring within the first minutes following exposure. Such reactions can develop both in patients without documented history of allergy as well as in patients with pre-existing allergic conditions or a positive family history of allergies [5].

The earliest reports of benzodiazepine hypersensitivity predominantly described dermatologic reactions including utricaria, angioedema, macular erythema, photoallergic reactions, hematoma-like rash accompanied by pruritus, erythema multiforme, and facial edema [5,9,27]. Cutaneus manifestations were reported in 13 out of 16 analyzed cases. More severe reactions involve the respiratory and cardiovascular systems. Respiratory symptoms were observed in 7 cases, whereas hemodynamic disturbances occurred in 10 out of 16 analyzed cases. The most severe complication is anaphylaxis, characterized by hypotension, decreased oxygen saturation, and reduced systolic blood pressure. Additional potential symptoms include bradycardia or tachycardia, bronchospasm, respiratory depression with wheezing, stridor, and use of additional respiratory muscles. Electrocardiographic abnormalities were reported in 4 cases, including QRS complex widening, ST-segment elevation and sinus bradycardia [5,13]. Paradoxical reactions, particulary to midazolam, were reported in 2 cases. Patients may present agitation, confusion, myoclonus, and anxiety. Common behavioral abnormalities include impulsivity, aggression, excessive talkativeness, and emotional instability. These reactions can occur shortly after administration of the drug as well as during emergence from general anesthesia. Symptoms of paradoxical reaction typically subside following flumazeni [23,24].

In most cases, the symptoms of hypersensitivity appear suddenly within a few minutes of benzodiazepine administration; however, symptoms occuring several hours later are also possible, including skin reactions, upper abdominal pain and nausea. In some cases, during an intraoperative hypersensitivity episode, tryptase levels were measured to assess mast cells activation [5]. Manifestation of hypersensitivity generally resolves after discontinuation of the offending drug. However, epinephrine may be required in the case of anaphylactic shock, and symptomatic treatment may include oxygen therapy, corticosteroids, short-acting beta-agonists, intravenous fluids, flumazenil, and antihistaminic drugs. Overall, clinical manifestation of benzodiazepine hypersensitivity is heterogeneous, non-specific, and can be easily mistaken for delirium, withdrawal, or the effects of other medications. Although, benzodiazepines are considered safe for premedication, the limited number of reported hypersensitivity reactions makes it difficult to identify clear risk factors in patients undergoing sedation [27].

We analyzed 16 case reports published between 2015 and 2025, and a summary of the clinical characteristics is presented in Table 3. The most frequent symptoms involved the cardiovascular system. Severe cardiovascular manifestations observed in reported cases were most likely a consequence to anaphylaxis rather than a direct cardiotoxic effect of benzodiazepines. These reactions were mainly mediated by excessive histamine release and other inflammatory mediators, resulting in vasodilatation and increased vascular permeability. Sometimes, reactions were difficult to manage and life-threatening, manifesting as hypotension refractory to vasopressors [12,28]. These symptoms were often accompanied by respiratory symptoms, consistent with observations in all analyzed original studies. Cutaneous manifestations were present in a significant proportion of reported cases cutaneous manifestations appeared, most often as urticaria, erythema, or edema of the head and neck tissues. However, cases of anaphylactic shock without cutaneous involvement were also documented [5,12,29,30].

In our analysis, IDT yielded positive results more frequently than SPT forbenzodiazepine hypersensitivity. When both tests were performed for the same drug, the intradermal test was positive in 7 cases, whereas the skin prick test was positive in 4 cases. However, the sensitivity and specificity of these two diagnostic methods for benzodiazepine hypersensitivity have not been evaluated in large cohort studies. Owing to the limited number of reported cases, these observations should be interpreted with caution and should not be considered evidence of diagnostic superiority of IDT over SPT. Serum tryptase served as a marker of mast cell degranulation, confirming anaphylaxis, regardless of whether the underlying mechanism was IgE-mediated or pseudoallergic.

### 2.5. Diagnostic Strategies

During the acute phase of a suspected hypersensitivity reaction, diagnostic assessment must be initiated promptly. Accurate recognition of anaphylaxis, based primarily on clinical presentation, is crucial. For this purpose, the patient can be assessed using a clinical scoring system that allocates points based on five elements: cardiovascular symptoms, respiratory symptoms, dermal or mucosal symptoms, the presence of combined symptoms, and the time from administration of the suspected drug to the onset of symptoms. The probability of a perioperative immediate hypersensitivity reaction (IHR) can then be estimated based on the total score obtained, ranging from unlikely to almost certain [39]. Emergency procedures should be implemented immediately, while blood samples for laboratory evaluation should be obtained in parallel. A detailed diagnostic investigation aimed at identifying the causative agent should be undertaken only after the patient’s clinical condition has stabilized. Supportive methods for diagnosing anaphylaxis include measurement of serum tryptase and histamine levels. These should be taken during the acute phase and again after around 24 h, once the episode has resolved [11,40,41]. Tryptase concentration should be measured during the acute reaction, ideally within 1–2 h of symptom onset, and compared with a baseline value obtained after recovery, as interpretation relies on the relative increase rather than comparison with a fixed reference range [15]. Elevated serum concentrations of tryptase and histamine indicate mast cell activation but do not distinguish between IgE-mediated and non-IgE-mediated mechanisms, as both pathways can result in mast cell degranulation. Therefore, these biomarkers support the diagnosis of anaphylaxis but do not, on their own, define the underlying immunological pathway [9]. Furthermore, a clinically significant increase in serum tryptase is not observed in all cases of anaphylaxis, which limits its diagnostic sensitivity [15].

Skin testing remains the standard diagnostic method used for perioperative drug hypersensitivity. Its primary role is to identify the allergen responsible for the reaction among the multiple substances administered during anesthesia and to evaluate potential cross-reactivity between drugs, thereby facilitating the selection of safe alternatives for future procedures. Diagnostic testing is generally delayed for several weeks after the reaction, as earlier assessment may yield false-negative results due to temporary mediator depletion in mast cells and basophils. The assessment of benzodiazepine hypersensitivity usually involves prick and intradermal skin tests [9,41]. These methods help identify the responsible agent among the multiple drugs administered during perioperative care and any excipients or auxiliary substances [11,14,40]. However, the diagnostic accuracy of skin testing may be limited not only by the timing of testing but also by the limited availability of validated concentrations for certain drugs. Moreover, negative results do not completely exclude hypersensitivity, and in selected cases a drug provocation test may be required to confirm tolerance. In this diagnostic approach, strict clinical supervision is essential, and the test should be performed in a setting equipped to provide immediate treatment in the event of an anaphylactic reaction [42].

In the differential diagnosis, hypersensitivity to other drugs administered during premedication must be considered. Neuromuscular blocking agents should be evaluated first, as they are the most common cause of perioperative anaphylaxis. Similarly, reactions ranging from mild symptoms to full-blown anaphylactic shock may be triggered by antibiotics, chlorhexidine, or latex, although any administered drug or substance could potentially be responsible [9,42]. Consequently, skin testing plays an important role in ensuring that all administered substances are appropriately evaluated. Cross-reactivity among benzodiazepines should also be considered during the diagnostic process, although it is not fully understood. A recent case report by Nakai et al. described a patient who experienced remimazolam-induced anaphylaxis after previous exposure to midazolam while remaining tolerant to another benzodiazepine, brotizolam. This suggests that cross-reactivity may vary among individual benzodiazepines and cannot be assumed across the class. This uncertainty poses diagnostic challenges, as testing other benzodiazepines after an anaphylactic reaction may be associated with significant risk [30].

### 2.6. Management and Prevention

The prevention and management of benzodiazepine-related hypersensitivity reactions rely on the prompt recognition of clinical manifestations in the perioperative setting and the rapid initiation of appropriate treatment. When anaphylaxis is suspected, immediate administration of epinephrine remains the first-line intervention because of its rapid hemodynamic and bronchodilatory effects. In reported perioperative cases, additional supportive measures have included vasopressors (e.g., ephedrine or phenylephrine), aggressive fluid resuscitation, and supplemental oxygen to stabilize cardiovascular and respiratory function.

Adjunctive therapies such as corticosteroids, antihistamines, and β_2_-agonists may be administered to mitigate persistent symptoms. However, in the context of suspected benzodiazepine-induced reactions, particular attention should be paid to the identification of the causative agent among multiple perioperative drugs, which often requires a structured diagnostic approach.

Post-event evaluation with in vitro assays and/or allergy testing is essential to confirm the diagnosis and to guide future anesthetic planning, thereby preventing inadvertent re-exposure. Documentation of the reaction and clear communication with the patient and healthcare providers are critical components of long-term prevention.

In addition, the use of multimodal or combined anesthetic techniques may reduce the need for benzodiazepines in susceptible individuals, thereby minimizing the risk of recurrent hypersensitivity reactions in subsequent procedures [14,29,36,38,43].

## 3. Discussion

Although benzodiazepines are generally considered safe drugs, rare cases of hypersensitivity reactions have been described in individual cases, most often associated with midazolam, including both IgE-mediated anaphylaxis and non-immune-mediated anaphylactoid reactions. Evidence regarding these reactions is largely limited to case reports and small observational studies. Data from the 6th National Audit Project (NAP6), conducted by the Royal College of Anaesthesia, identified 199 confirmed agents responsible for perioperative anaphylaxis. These were most frequently attributed to antibiotics (94/199), neuromuscular block agents (65/199), and chlorhexidine (18/199), whereas benzodiazepines were not identified as causative agents [44]. These findings indicate that they are rarely implicated in large-scale epidemiological studies, although under-recognition or misattribution cannot be excluded. Furthermore, 16 cases of confirmed benzodiazepine hypersensitivity have been reported in the literature. Nevertheless, the limited number of reported cases and their heterogeneous clinical manifestation preclude meaningful epidemiological comparison with other premedication drugs, such as opioids or neuromuscular blocking agents. Despite their rarity, benzodiazepine hypersensitivity remains a clinical concern, posing challenges for perioperative premedication and procedural safety. Clinical presentation of hypersensitivity is heterogenous, particularly when multiple drugs are co-administered, as overlapping effects may obscure recognition of benzodiazepine-related reactions [30]. Potential cross-reactivity between benzodiazepines should also be considered. Structural similarities between midazolam and ramimazolam suggest a theoretical possibility of cross-reactions. However, structural resemblance exclusively does not provide evidence for shared antigenic determinants. Case reports describing safe administration of midazolam both before and after ramimazolam-induced hypersensitivity further argue against a widespread or universal pattern of cross-reactivity between these agents. However positive intradermal skin tests to both drugs have been reported. Although such findings may suggest potential cross-reactivity, the limitations of skin tests and the challenges associated with their interpretation must be carefully acknowledged. These include the lack of standardized testing protocols and the uncertain diagnostic performance of these tests, as well as the risk of irritant or false-positive reactions. Moreover, the limited number of published reports describing such cases prevents a conclusive evaluation of the frequency and clinical significance of cross-reactivity. Additionally, the lack of specific immune markers makes it difficult to unambiguously determine the underlying mechanism of these reactions. Available evidence suggests that they may involve both immunologic and non-immunologic mechanisms [5]. The diagnosis is often delayed, because there are no clear criteria for the immediate identification of hypersensitivity reactions. The 2023 anaphylaxis practice parameter update from the American College of Allergy, Asthma & Immunology and American Academy of Allergy, Asthma & Immunology recommends measuring serum tryptase as soon as possible after suspected anaphylaxis, preferably within two hours of symptom onset, with a subsequent measurement to determine whether the initial elevation was clinically significant [45]. However, other research indicates that tryptase testing is performed in only 34–67% of patients with perioperative anaphylaxis, regardless of the causative agent [46]. This suggests that delayed diagnosis and inconsistent tryptase measurement represent a broader systemic issue rather than a limitation specific to benzodiazepine-induced reactions. Moreover, comprehensive diagnostic evaluation, including skin tests, is typically performed once the patient is clinically stable. There is limited research, few case reports, and no well-defined criteria to accurately diagnose hypersensitivity [9].

Allergological assessment and pharmacogenetic evaluation may help identify patients at increased risk of benzodiazepine hypersensitivity. Skin tests and drug provocation tests constitute important components of the diagnostic work-up for hypersensitivity; however, their use is associated with several limitations. Provocation tests may elicit severe IgE-mediated as well as non-IgE-mediated reactions; therefore, they should be performed only under close medical supervision and in appropriately controlled clinical settings.

There are scientific studies investigating the polymorphism of genes encoding GABA-A receptors and enzymes involved in benzodiazepine metabolism. Hypothetically, such genetic variability could contribute to individual differences in susceptibility to hypersensitivity reactions, as well as in sedative response. However, there is currently no direct evidence linking specific genetic variants with confirmed benzodiazepine hypersensitivity [47,48]. Therefore, this area remains largely speculative and represent a potential direction for future research.

Current evidence regarding benzodiazepine hypersensitivity is based predominantly on case reports and small-sample retrospective analyses. Although these studies are precious for identifying safety signals, their statistical strength remains limited. The lack of control groups and significant methodological variability—including differences in administration route, dosage, patient populations, diagnostic criteria, and allergy assessment—substantially limit cross-study comparability. Consequently, the available data do not permit a reliable estimation of the prevalence of benzodiazepine hypersensitivity in either the general population or in susceptible groups. In addition, the current evidence does not allow for definitive conclusions regarding the pathophysiological mechanism underlying benzodiazepine hypersensitivity. These constraints impede the identification of predisposing factors and, as a result, the current state of knowledge does not support the development of clear, evidence-based clinical recommendations and standardized diagnostic strategies. These considerations highlight the need for further studies in large and diverse patient populations, as well as analyses of a greater number of cases. Detailed allergy work-ups could help determine the true frequency of reactions, identify risk factors, and develop standardized diagnostic criteria. Such efforts will ultimately support the creation of guidelines aimed at enhancing the safety of patients with benzodiazepine hypersensitivity during medical procedures.

## 4. Materials and Methods

### 4.1. Search Strategy

This study was conducted as a narrative review. The literature review process was conducted between 14 October 2025 and 30 November 2025. The literature screening and thematic evaluation were performed by seven reviewers with expertise in allergy and perioperative medicine, covering publications from 1 January 2000 to 30 November 2025. The following databases were searched: Google Scholar, PubMed, Scopus, Embase, and EBSCO. To manage the variability and large volume of Google Scholar results, a relevance filter was applied to prioritize the most pertinent publications, followed by manual screening of titles and abstracts to further select studies meeting the inclusion criteria. Duplicates were removed prior to full-text evaluation.

No other database filters or restrictions were applied to maximize the sensitivity of the search strategy and ensure comprehensive thematic coverage. To maintain specificity and reproducibility, despite the broad search, all titles, abstracts, and keywords retrieved from all databases were manually screened to identify potentially relevant publications, and irrelevant or clearly off-topic articles were excluded at this stage.

To comprehensively explore the subject of benzodiazepine hypersensitivity, the following search terms were used in various combinations: “Benzodiazepines” (including specific agents such as “midazolam” and “remimazolam”) AND (“Hypersensitivity mechanisms” OR “Premedication” OR “Diagnostics”).

Additionally, reference lists of selected articles were manually screened to identify further relevant publications not captured during the initial database search.

### 4.2. Eligibility Criteria

The narrative review included original research articles, case reports, case series, review articles, textbook chapters, and expert recommendations published between January 2000 and November 2025. The scope of inclusion covered data concerning the incidence of benzodiazepine hypersensitivity, clinical manifestations, immunological mechanisms, diagnostic strategies, treatment principles, and prevention of hypersensitivity reactions.

Owing to the clinical significance of the four-stage perioperative anaphylaxis severity scale developed by Ring and Messmer, one seminal publication published outside the predefined time frame was also included.

Priority was given to articles published in peer-reviewed journals, studies conducted in humans, publications written in English, and studies directly related to the topic of benzodiazepine hypersensitivity. Review articles and textbook chapters were included mainly to provide background and context and to summarize evidence from primary studies, rather than to serve as original data sources, minimizing overlap with primary research.

Publications were excluded if they consisted solely of conference abstracts, letters to the editor, non-peer-reviewed materials, animal studies, or articles not directly relevant to the subject of this review.

### 4.3. Study Selection and Data Synthesis

The study selection process followed a structured approach within the flexible framework of narrative review methodology. Specifically, structured elements included the use of predefined inclusion and exclusion criteria and independent screening by seven reviewers, while flexibility was maintained in the interpretation of thematic relevance and synthesis of evidence. Initial screening was based on titles and abstracts, and articles deemed potentially relevant were subsequently assessed in full text. In the case of uncertainty regarding thematic relevance, full-text analysis was conducted before making a final inclusion decision. Discrepancies or disagreements among reviewers were resolved through discussion and consensus.

This assessment was performed qualitatively as part of the narrative synthesis, focusing on the appropriateness of study design, the size and characteristics of study populations, the clarity of diagnostic methods, completeness of reported results, potential sources of bias, and the consistency of conclusions with the presented data.

A total of 49 publications were included in the final analysis.

This narrative synthesis was designed to provide a comprehensive and clinically relevant overview of benzodiazepine hypersensitivity, integrating available evidence with expert-informed interpretation while maintaining methodological transparency.

## 5. Conclusions

Based on the available literature, hypersensitivity reactions to benzodiazepines have been reported primarily in case reports and small case series, indicating that the overall level of evidence remains limited. Although such reactions appear to be rare, they are clinically relevant owing to their potential to cause severe and life-threatening anaphylactic episodes. Among the reported cases, IgE-mediated type I hypersensitivity mechanisms are the most frequently described. However, the small number of cases and the lack of robust supporting evidence substantially limit the strength of these observations. Isolated cases of delayed type IV reactions and non-immunologic mechanisms, including pseudoallergic reactions, have also been described, particularly in association with midazolam.

The heterogeneity of the reported mechanisms highlights the diagnostic challenges encountered in clinical practice. Initiating diagnostic evaluation during the acute phase, followed by further assessment after patient stabilization, may help reduce the risk of recurrent reactions. However, the absence of standardized diagnostic pathways limits consistency in clinical management.

Despite the availability of several diagnostic approaches, a universally accepted gold standard for the diagnosis and management of benzodiazepine hypersensitivity has not yet been established. This limitation underscores the need for well-designed clinical studies to clarify the underlying mechanisms and to support the development of standardized diagnostic and therapeutic strategies. Future research should focus on the establishment of prospective registries to enable systematic data collection, the implementation of standardized allergy diagnostic protocols, and detailed pathophysiological investigations aimed at distinguishing immune-mediated reactions from non-immune mechanisms.

In summary, although hypersensitivity reactions to benzodiazepines appear to be uncommon, their potentially severe clinical course warrants continued clinical vigilance. Increased clinician awareness, systematic diagnostic evaluation, and further research are essential to improve patient safety and support the safe use of benzodiazepines in clinical practice.

## Figures and Tables

**Table 1:** Ring and Messmer perioperative anaphylaxis four-step grading scale [19]. The table was independently developed by the authors.

Clinical Signs	Grade
Skin symptoms and/or mild fever reaction	I
Measurable, but not life-threatening Cardiovascular reaction (tachycardia, hypotension) Gastrointestinal disturbance (nausea) Respiratory disturbance	II
Shock, life-threatening spasm of smooth muscles (bronchi, uterus, etc.)	III
Cardiac or respiratory arrest	IV

**Table 2:** Review of original studies on allergic reactions to benzodiazepines; SPT—Skin Prick Test, ID—Intradermal Test, BAT—Basophil Activation Test, BDZ—Benzodiazepines. The table was independently developed by the authors.

Investigated BDZ	Diagnostic Method	Population	Study Type	Author
Midazolam	SPT, ID, BAT, serological tests	14 adults	Original research	Eberlein et al.
Midazolam	SPT, ID	459 adults	Prospective study	Beyaz et al.
Midazolam	SPT, ID, BAT	145 adults	Retrospective study	Liu et al.
Midazolam	SPT, ID, provocation tests	101 adults	Retrospective study	D’Onofrio et al.
Midazolam	SPT, ID, serological tests	50 children	Retrospective study	Karaatmaca et al.

**Table 3:** Reported cases of immunological and non-immunological hypersensitivity reactions to benzodiazepines in the perioperative procedures (2015–Present); SPT—Skin Prick Test; IDT—Intradermal Tests; AML—Acute Myeloid Leukemia. The table was independently developed by the authors.

Possible Mechanism	Laboratory Findings	Case Description	Age/Sex	Author (Year)
Anaphylactoid reaction (non IgE-mediated) to midazolam	Serum tryptase: Elevated (16.7 μg/L) Troponin: Increased (0.151 to 0.9 ng/dL)	During preoperative preparation before transurethral prostatectomy, the patient received IV midazolam (2 mg). Immediately, he developed ST depression on ECG, hypotension (80/45 mmHg), and urticaria on the legs and back. The patient’s condition progressed to angina, dyspnea, and sweating. Emergency coronary angiography revealed Kounis syndrome (allergic myocardial infarction secondary to the acute hypersensitivity to midazolam).	70/Male	Ateş et al. (2015) [31]
Anaphylactoid reaction (non IgE-mediated) to midazolam probable direct histamine release	Multiple allergy tests, including skin tests, revealed no clear evidence of an allergic reaction.	Patient after the full induction dose of IV midazolam (5 mg) before mandible tumor resection developed a life-threating reaction: hypotension (systolic BP 65 mmHg), bronchospasm, ST depression in ECG, widespread flush, and edema of the eyes. She had a history of skin reactions to antibiotics and CT contrast.	50/Female	Ayuse et al. (2015) [32]
IgE-dependent hypersensitivity (type I) to midazolam likely mediated by a hapten-carrier complex (positive IDT and persistence of skin reactivity)	Specific IgE 1.93% (nonspecific binding (0.7%) SPT (midazolam): Negative IDT (midazolam): Positive After 1 year: Specific IgE 0.66% (nonspecific binding 0.41%) IDT: Positive	A patient with a history of allergic asthma, rhinitis, and a family history of asthma was admitted for adenoidectomy. After premedication with oral midazolam (0.5 mg/kg), he developed dyspnea, wheezing, and prolonged expiration with SpO_2_ < 90%.	7/Male	Bernardini et al. (2017) [29]
IgE-dependent anaphylaxis (type I hypersensitivity) to midazolam, probable hapten–carrier complex mechanism	Serum Tryptase: Elevated (31.7 μg/L) SPT and IDT (midazolam): Positive	The patient received IV midazolam (2 mg) followed by induction drugs (including vecuronium) before EP ablation. Ten minutes after re-dose of vecuronium, his BP dropped to 68/24 mmHg, HR 125 bpm and hives appeared on the chest and face.	17/Male	Landsem et al. (2017) [6]
Undetermined reaction to midazolam Type 1—positive IDT Pseudoallergic—negative SPT and IgE	Specific IgE: Negative SPT (midazolam): Negative IDT (midazolam, ketamine): Positive	A patient diagnosed with nephrolithiasis developed generalized urticaria immediately after IV midazolam and other anesthetics. Similar reactions happened before. The reaction was limited to the skin (no systemic symptoms) and resolved quickly after treatment.	4/Female	Arikan Ayyildiz et al. (2018) [33]
Anaphylaxis (type I hypersensitivity) to midazolam	Not mentioned	A patient with AML was scheduled for bone marrow aspiration and lumbar puncture. Despite 7 prior exposures to midazolam without incident, this time, immediately after IV midazolam (5 mg) administration, he developed dyspnea. Vomiting, chest pain, hypotension (80/40 mmHg), urticaria, and edema of face and neck.	17/Male	Çakmakcı et al. (2018) [34]
Delayed hypersensitivity reaction (type IV)—fixed drug eruption to lorazepam	Patch test (lorazepam): Negative Controlled oral challenge test (lorazepam): Positive (pruritus and lesions appeared 30 min after 1 mg cumulative dose) Peripheral eosinophilia: 10%	A patient developed cutaneous pruritus and macular lesions on the trunk and limbs during hospitalization after bladder surgery (post-operative period). The reaction recurred 8 h after taking oral lorazepam. Lesions healed with residual hyperpigmentation.	68/Male	Agulló-García et al. (2018) [35]
IgE-dependent hypersensitivity (type I anaphylaxis) to midazolam with possible midazolam-specific IgE antibodies	IDT (9 weeks after reaction): Positive	A patient was preparing for endoscopic discectomy under anesthesia. She had previously received midazolam without incident. This time, one minute after IV midazolam administration (1 mg), she developed BP 41/30 mmHg, HR 46 bpm, bronchospasm (wheezing, obstructive capnography), and skin erythema.	62/Female	Jeon et al. (2019) [36]
IgE-dependent hypersensitivity (type I anaphylaxis) to remimazolam	After onset: Serum tryptase: Normal (5.8 μg/mL) Histamine: Elevated (1.5 ng/mL) IDT (remimazolam midazolam): Positive	A patient with no history of allergy experienced facial flushing, hypoxia, hypotension (BP 49 mmHg), and laryngeal edema 2 min after IV remimazolam administration.	32/Male	Tsurumi et al. (2021) [13]
IgE-dependent anaphylaxis (type I hypersensitivity) to midazolam most likely because of likely hapten–carrier complex formation	Specific IgE: Negative SPT (midazolam): Negative IDT (midazolam): Positive	A patient was undergoing elective video laparoscopic cholecystectomy with IV midazolam (2 mg) administration as premedication. A few minutes after induction, he developed bradycardia (35 bpm). Wide QRS, ST elevation, asystole, and ventricular fibrillation.	54/Male	Nucera et al. (2021) [27]
IgE-dependent anaphylaxis (type I hypersensitivity) to remimazolam with IgE mediated cross-reactivity	IDT (midazolam): Positive IDT (remimazolam): Negative	A patient with no history of allergy was premedicated by IV remimazolam (10 mg) before colonoscopy. He developed stridor, erythema of the face, neck, and chest, swelling of the eyelids and lips, hypotension (BP 77/47 mmHg), epiglottic edema, and diarrhea.	41/Male	Hu et al. (2023) [14]
IgE-dependent anaphylaxis (type I hypersensitivity) to midazolam	Serum tryptase: Elevated (56.5 μg/mL) SPT (midazolam): Positive IDT (midazolam): Positive	A patient with multiple comorbidities and prior exposure to midazolam before incident was premedicated by midazolam IV (2 mg) before hydrocelectomy. Seven minutes after spinal anesthesia, he developed hypotension refractory to vasopressors (38/28 mmHg), HR 31 bpm, and Sp02 86%.	73/Male	Winegarner et al. (2023) [12]
Non-immunological (pseudoallergic) reaction to remimazolam	Serum tryptase: Normal (3.9 μg/L) IDT (all drugs given including remimazolam): Negative Provocation test (remimazolam): Positive	A patient with a history of topical anesthetic allergy (presumed lidocaine) after administration IV remimazolam (10 mg) developed hypotension (32/18 mmHg), tachycardia (102 bpm), and a generalized skin rash with swelling after IV remimazolam (10 mg) administration.	51/Female	Lee et al. (2023) [15]
Delayed hypersensitivity reaction (type IV) to midazolam	Latex IgE and serum complement levels: Normal SPT and IDT (midazolam): Positive	A patient underwent salpingoophorectomy. Six hours after anesthesia induction, she developed unilateral tongue swelling, which persisted for three weeks despite treatment.	30/Female	Tejada et al. (2024) [37]
Undetermined reaction to midazolam and remimazolam	Acute tryptase: Elevated (9.2 μg/L) SPT and IDT (remimazolam, midazolam): Not performed, because it was considered too risky	A patient with a history of two severe reactions (during FFP and anesthesia) where midazolam was the sole drug used during both procedures. During gastrectomy, the patient developed tracheal and bronchial edema, bronchospasm, hypotension, and cardiac arrest.	75/Male	Nakai et al. (2024) [30]
IgE-dependent anaphylaxis (type I hypersensitivity), with two possible causative agents—midazolam and patent blue dye	Serum tryptase: Elevated SPT (midazolam, Patent Blue Dye): Positive IDT (midazolam, Patent Blue Dye): Positive	A woman with no history of allergy was undergoing left total mastectomy. Five minutes after induction IV midazolam and other anesthetic drugs and injection of patent blue dye, she developed MAP 25–45 mmHg and bradycardia (40 bpm). The reaction was refractory to vasopressors. Twenty minutes after the collapse, a diffuse skin rash and edema appeared.	30/Female	Lagarteira et al. (2025) [38]

## Data Availability

No new data were created or analyzed in this study. Data sharing is not applicable.

## References

[1] Simić D., Stanković Z., Stević M., Petrov-Bojičić I. (2023). Oral premedication with benzodiazepines. Galen. Med. J..

[2] Marsh A., McIndoe A.K. (2004). Premedication. Anaesth. Intensive Care Med..

[3] Salud Navarra. https://www.navarra.es/home_en/Temas/Portal+de+la+Salud/Profesionales/Documentacion+y+publicaciones/Publicaciones+tematicas/Medicamento/BIT/Vol+22/DTB+Vol+22+N+2.htm.

[4] Silva A.C.D., Boralli C.F., Portilho N.C., Garro L.S., Ribeiro M.R., De Magalhaes M.C., Campos L., Motta A.A., Filho J.E.K., Giavina-Bianchi P. (2018). Midazolam is a major cause of intraoperative immediate hypersensitivity reactions. J. Allergy Clin. Immunol..

[5] Nucera E., Parrinello G., Buonomo A., Aruanno A., Rizzi A. (2021). Allergic reactions to midazolam: A case series from an Italian allergy unit. Allergol. Immunopathol. (Madr)..

[6] Landsem L.M., Ross F.J., Eisses M.J. (2017). A case of midazolam anaphylaxis during a pediatric patient’s first anesthetic. J. Clin. Anesth..

[7] Laguna J., Archilla J., Doña I., Corominas M., Gastaminza G., Mayorga C., Berjes-Gimeno P., Tornero P., Martin S., Planas A. (2018). Practical guidelines for perioperative hypersensitivity reactions. J. Investig. Allergol. Clin. Immunol..

[8] Benzodiazepiny. https://www.mp.pl/interna/chapter/B16.II.20.4.

[9] Haybarger E., Young A.S., Giovannitti J.A. (2016). Benzodiazepine allergy with anesthesia administration: A review of current literature. Anesth. Prog..

[10] Bieńkowski P., Samochowiec J., Sienkiewicz-Jarosz H., Wichniak A., Mastalerz-Migas A. (2019). Bezpieczne stosowanie benzodiazepin w podstawowej opiece zdrowotnej—Rekomendacje dla lekarzy rodzinnych. Lek. POZ.

[11] Hasushita Y., Nagao M., Miyazawa Y., Yunoki K., Mima H. (2022). Cardiac arrest following remimazolam-induced anaphylaxis: A case report. A&A Pract..

[12] Winegarner A., Kendall M.C., Stephen M., Siddiqui A. (2023). Delayed anaphylactic reaction to midazolam in the absence of immediate respiratory or skin manifestations. Case Rep. Anesthesiol..

[13] Tsurumi K., Takahashi S., Hiramoto Y., Nagumo K., Takazawa T., Kamiyama Y. (2021). Remimazolam anaphylaxis during anesthesia induction. J. Anesth..

[14] Hu X., Tang Y., Fang X. (2023). Laryngeal edema following remimazolam-induced anaphylaxis: A rare clinical manifestation. BMC Anesthesiol..

[15] Lee S., Park J., Kim N.H., Hong H., Sohn K.H., Kang H.Y., Kim M.K., You A.H. (2023). Remimazolam anaphylaxis during induction of general anesthesia confirmed by provocation test—A case report and literature review. Medicina (Kaunas).

[16] Pallardy M., Bechara R., Whritenour J., Mitchell-Ryan S., Herzyk D., Lebrec H., Merk H., Gourley I., Komocsar W.J., Piccotti J.R. (2024). Drug hypersensitivity reactions: Review of the state of the science for prediction and diagnosis. Toxicol. Sci..

[17] Pavlos R., Mallal S., Ostrov D., Buus S., Metushi I., Peters B., Phillips E. (2015). T-cell–mediated hypersensitivity reactions to drugs. Annu. Rev. Med..

[18] Pichler W.J. (2019). Immune pathomechanism and classification of drug hypersensitivity. Allergy.

[19] Ring J., Messmer K. (1977). Incidence and severity of anaphylactoid reactions to colloid volume substitutes. Lancet.

[20] Gomez A.M., Gonzalez M.B., Alvarez N.C., Muñoz M.G., Sastre V.H., Arceo J.P., Induráin B.V. (2015). Perioperative anaphylactic reactions: Review and procedure protocol in paediatrics. Allergol. Immunopathol. (Madr)..

[21] Liu X., Gong R., Xin X., Zhao J. (2022). Clinical characteristics and allergen detection of perioperative anaphylaxis: A 12-year retrospective analysis from an anesthesia clinic in China. Perioper. Med..

[22] Karaatmaca B., Sahiner U.M., Sekerel B.E., Soyer O. (2021). Perioperative hypersensitivity reactions during childhood and outcomes of subsequent anesthesia. Paediatr. Anaesth..

[23] Park S., Ibrahim M., Torres A. (2024). Persistent paradoxical reaction to midazolam despite general anesthesia with dexmedetomidine. Case Rep. Anesthesiol..

[24] Sivakumar S., Mendonca R., Demeterio D., Girshin M. (2021). Paradoxical reactions to midazolam in a term parturient after intravenous sedation during cesarean section. Cureus.

[25] Jin E.H., Song J.H., Lee J., Bae J.H., Chung S.J. (2021). Midazolam dose is associated with recurrence of paradoxical reactions during endoscopy. World J. Clin. Cases.

[26] Zhao Z., Liu L., Zhang X. (2023). Remimazolam-induced paradoxical reaction in a bipolar patient: A case report. Clin. Case Rep..

[27] Nucera E., Aruanno A., Buonomo A., Parrinello G., Rizzi A. (2021). Hypersensitivity reaction to midazolam: A case of cardiorespiratory failure. Postepy Dermatol. Alergol..

[28] Nguyen S.M.T., Rupprecht C.P., Haque A., Pattanaik D., Yusin J., Krishnaswamy G. (2021). Mechanisms governing anaphylaxis: Inflammatory cells, mediators, endothelial gap junctions and beyond. Int. J. Mol. Sci..

[29] Bernardini R., Bonadonna P., Catania P., Passalacqua G. (2017). Perioperative midazolam hypersensitivity in a seven-year-old boy. Pediatr. Allergy Immunol..

[30] Nakai T., Kako E., Ota H., So M.H., Sobue K. (2024). Remimazolam anaphylaxis in a patient not allergic to brotizolam: A case report and literature review. BMC Anesthesiol..

[31] Ateş A.H., Kul S. (2015). Acute coronary syndrome due to midazolam use: Kounis syndrome during a transurethral prostatectomy. Turk Kardiyol. Dern. Ars..

[32] Ayuse T., Kurata S., Ayuse T. (2015). Anaphylactoid-like reaction to midazolam during oral and maxillofacial surgery. Anesth. Prog.

[33] Ayyildiz Z.A., Işık S., Sözmen Ş.Ç., Tezcan D., Karaman Ö., Uzuner N. (2018). Midazolam and ketamine hypersensitivity in a four-year-old child. Asthma Allergy Immunol..

[34] Çakmakcı S., Bayhan T., Cihan M.K., İlhan İ.E. (2018). Anaphylaxis with midazolam in pediatric hematology-oncology unit: A case report. Turk Pediatri Ars..

[35] Agulló-García A., Garcés Sotillos M., Colás Sanz C. (2018). Fixed drug eruption due to lorazepam. J. Investig. Allergol. Clin. Immunol..

[36] Jeon Y.S., Shim J., Jun E.H., Choi S.T., Jung H.S. (2019). Midazolam anaphylaxis during general anesthesia: A case report. Medicine (Baltimore).

[37] Tejada N., Vu A. (2024). Midazolam-induced perioperative angioedema: A case report. Ann. Allergy Asthma Immunol..

[38] Lagarteira B., Flor de Lima M., Bento M., Santa C., Rego J. (2025). Unraveling the cause of perioperative anaphylaxis: The role of patent blue dye and midazolam. Cureus.

[39] Hopkins P.M., Cooke P.J., Clarke R.C., Guttormsen A.B., Platt P.R., Dewachter P., Ebo D.G., Garcez T., Garvey L.H., Hepner D.L. (2019). Consensus clinical scoring for suspected perioperative immediate hypersensitivity reactions. Br. J. Anaesth..

[40] Funabiki R., Horiuchi T., Shiraishi T., Orihara M., Nagumo K., Saito S. (2025). Anaphylaxis due to midazolam administered before induction of general anesthesia: A case report. JA Clin. Rep..

[41] Takazawa T., Yamaura K., Hara T., Yorozu T., Mitsuhata H., Morimatsu H. (2021). Practical guidelines for the response to perioperative anaphylaxis. J. Anesth..

[42] Admass B.A., Hassen A.E., Agegnehu A.F., Temesgen M.M., Gebeyehu N.A., Ferede Y.A., Tegegne B.A. (2023). Management of perioperative anaphylaxis: Systematic review. Int. J. Surg. Open.

[43] Di Leo E., Delle Donne P., Calogiuri G.F., Macchia L., Nettis E. (2018). Focus on the agents most frequently responsible for perioperative anaphylaxis. Clin. Mol. Allergy.

[44] Harper N.J.N., Cook T.M., Garcez T., Farmer L., Floss K., Marinho S., Torevell H., Warner A., Ferguson K., Hitchman J. (2018). Anaesthesia, surgery, and life-threatening allergic reactions: Epidemiology and clinical features of perioperative anaphylaxis in the 6th National Audit Project (NAP6). Br. J. Anaesth..

[45] Golden D.B.K., Wang J., Waserman S., Akin C., Campbell R.L., Ellis A.K., Greenhawt M., Lang D.M., Ledford D.K., Lieberman J. (2024). Anaphylaxis: A 2023 practice parameter update. Ann. Allergy Asthma Immunol..

[46] Misbah S.A., Krishna M.T. (2019). Peri-operative anaphylaxis—An investigational challenge. Front. Immunol..

[47] Fukasawa T., Suzuki A., Otani K. (2007). Effects of genetic polymorphism of cytochrome P450 enzymes on the pharmacokinetics of benzodiazepines. J. Clin. Pharm. Ther..

[48] Kosaki Y., Nishizawa D., Hasegawa J., Yoshida K., Ikeda K., Ichinohe T. (2024). γ-Aminobutyric acid type A receptor β1 subunit gene polymorphisms are associated with the sedative and amnesic effects of midazolam. Mol. Brain.

